# Identification and characterization of sequence signatures in the *Bacillus subtilis* promoter P_*ylb*_ for tuning promoter strength

**DOI:** 10.1007/s10529-019-02749-4

**Published:** 2019-11-05

**Authors:** Jiangtao Xu, Xiaoqing Liu, Xiaoxia Yu, Xiaoyu Chu, Jian Tian, Ningfeng Wu

**Affiliations:** Biotechnology Research Institute, Chinese Academy of Agricultural Sciences, Beijing, 100081 China

**Keywords:** *Bacillus subtilis*, Promoter, Random-scanning mutant, P_*ylb*_

## Abstract

**Objective:**

To thoroughly characterize the P_*ylb*_ promoter and identify the elements that affect the promoter activity.

**Result:**

The sequences flanking the − 35 and − 10 box of the P_*ylb*_ promoter were divided into six segments, and six random-scanning mutant promoter libraries fused to an enhanced green fluorescent protein EGFP were made and analyzed by flow cytometry. Our results showed that the four nucleotides flanking the − 35 box could mostly influence the promoter activity, and this influence was related to the GC content. The promoters mutated in these regions were successfully used for expressing the gene *ophc2* encoding organophosphorus hydrolase (OPHC2) and the gene *katA* encoding catalase (KatA).

**Conclusion:**

Our work identified and characterized the sequence signatures of the P_*ylb*_ promoter that could tune the promoter strength, providing further information for the potential application of this promoter. Meanwhile, the sequence signatures have the potential to be used for tuning gene expression in enzyme production, metabolic engineering, and synthetic biology.

**Electronic supplementary material:**

The online version of this article (10.1007/s10529-019-02749-4) contains supplementary material, which is available to authorized users.

## Introduction

*Bacillus subtilis* has been developed as a convenient host for the production of heterologous proteins and industrial enzymes (van Dijl and Hecker 2013). As the promoter is one of the key factors for efficiently expressing heterologous proteins (Blazeck and Alper 2013), a series of native promoters from *B*. *subtilis* have been identified and successfully used in *B*. *subtilis* for gene expression, such as the promoter P43 (Zhang et al. 2005), P_*xylA*_ (Bhavsar et al. 2001), P_*sacB*_ (Steinmetz et al. 1985) and P_*glv*_ (Ming et al. 2010). However, more endogenous promoters need to be explored for producing heterologous proteins.

Meanwhile, efforts have been made to enhance promoter strength. The most conventional strategy is modifying moderately conserved sequences of the promoter, such as the UP element, the core region (− 35 and − 10 motifs), and the 16 region (TRTG motif) within existing promoters (Cheng et al. 2016; Guan et al. 2016; Lee et al. 2010; Phan et al. 2012, 2015; Zhou et al. 2019). Furthermore, mutagenesis of the spacer region between the − 35 and − 10 regions in *E. coli* and *Lactobacillus (Lb.) plantarum* has successfully created high-coverage synthetic promoter libraries (De Mey et al. 2007). Saturation mutagenesis of a *Lactococcus lactis* promoter drastically modulates expression (Jensen and Hammer 1998). In addition, randomization of the spacer region of P_*ymdA*_ and P_*serA*_ in *B*. *subtilis* has generated mostly strong to medium promoters (Guiziou et al. 2016). Thus, mutagenesis of the spacer region is considered a rational methodology to modify prokaryotic promoter strength.

Previously, we had identified a highly efficient stationary phase promoter P_*ylb*_ in *B*. *subtilis*. The P_*ylb*_ promoter could induce higher levels of expression of active β-galactosidase, EGFP, RFP, pullulanase, and organophosphorus hydrolase from the late log phase to the stationary phase than that of the widely used P43 promoter, demonstrating strong potential to be used for the overexpression of useful proteins in *B*. *subtilis*. By scanning site-directed mutagenesis on the P_*ylb*_ promoter, we found that not only the − 35 and − 10 regions, but also the regions flanking the − 35 and − 10 regions were directly involved in the transcriptional activity of P_*ylb*_ (Yu et al. 2015). In this study, we focused on the flanking sequences to thoroughly characterize the P_*ylb*_ promoter and identify the elements that affect the promoter activity. Our work clarified the effect of the identified promoter sequence signatures on the activity of the P_*ylb*_ promoter. Meanwhile, the promoters libraries with the mutation in the sequence signatures have a wide range of activities, and have the potential to be used for tuning gene expression in enzyme production, metabolic engineering, and synthetic biology.

## Materials and methods

### Bacterial strains, plasmids and growth conditions

The bacterial strains and plasmids used in this study are listed in Supplementary Table 1. The *Escherichia coli* strain DH5α was used as the host for gene cloning and the construction of the mutated promoter libraries. The *B*. *subtilis* strain WB600 was used for promoter screening and gene expression. All the strains were cultured in Luria–Bertani medium (LB) at 37 °C under constant shaking (200 rpm). The concentrations of antibiotics used for selection were as follow: 100 μg/mL ampicillin (Amp), 10 μg/mL kanamycin (Kan), 50 μg/mL tetracycline (Tet).

### Construction of P_ylb_ random-scanning mutagenesis libraries

**Fig. 1:**
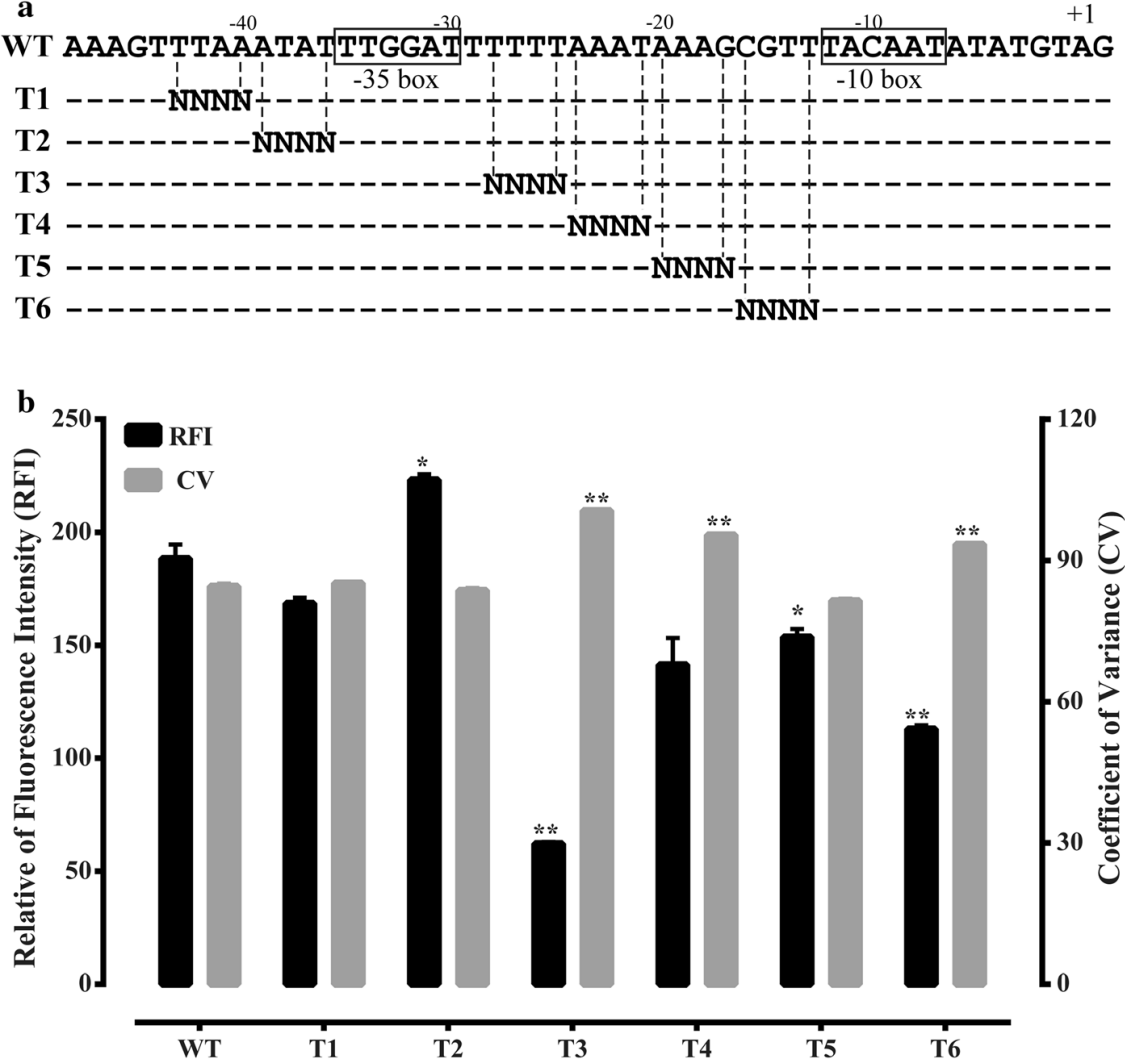
Construction and characterization of the P_*ylb*_ random-scanning mutagenesis libraries. **a** Schematic representation of the random-scanning mutagenesis target sites on the P_*ylb*_ promoter to construct the libraries. The promoter sequences were randomly mutagenized by the degenerate sequences within the corresponding primers. The − 35 and − 10 boxes are denoted by the open squares. The + 1 mark represents the transcription start site (TSS). **b** Fluorescence intensity output of mutated library promoters driving expression of *egfp* as determined by flow cytometry in triplicate. *p < 0.01; **p < 0.001, relative to the WT control

Six P_*ylb*_ random-scanning mutagenesis libraries were constructed by randomly mutating the four nucleotides in six segments, which located in the P_*ylb*_ region from position − 43 to − 10 (relative to TSS) (Fig. 1a). First, using the plasmid P_*ylb*_-G-P43-R-pUBC19 in which the promoter P_*ylb*_ controls the transcription of the reporter gene *egfp* encoding enhanced green fluorescent protein EGFP as the template, six mutant promoter libraries were generated by PCR with six degenerate primers P16-F-1, -2, -3, -4, -5 and -6 and the reverse primer V-R (Supplementary Table 2), the linear vector fragments were amplified with the forward primers P16-Fr-1, -2, -3, -4, -5 and -6 and the same reverse primer V-F (Supplementary Table 2). Following, the gel-purified promoter and vector fragments were assembled through POE-PCR (You and Percival Zhang 2012). The reaction solution had a total volume of 50 μL and contained 0.2 mM dNTP, 400–500 ng promoter fragment, the vector fragment (equimolar with the promoter fragment), 1.5 μL DMSO and 0.04 U/μL NEB high-fidelity Phusion DNA polymerase. The POE-PCR conditions were set as follows: 98 °C for 30 s, followed by 30 cycles of 10 s at 98 °C, 30 s at 55 °C, and 4.5 min at 72 °C, with a final extension step at 72 °C for 10 min. The POE-PCR products were isopropanol precipitated, resuspended in 10 μL ddH_2_O and then transformed into competent *E. coli* DH5α cells. All the *E. coli* transformants were collected together with sterile ddH_2_O and the plasmids from these cells were extracted by the TIANprep Mini Plamid Kit II (Tiangen Biotech, Beijing, China). Lastly, the plasmids of these P_*ylb*_ mutant libraries were transformed into the *B. subtilis* WB600 strain (Spizizen 1958) to construct the corresponding random-scanning mutagenesis libraries T1–T6.

### Characterization of promoter libraries by flow cytometry

To measure the activity of the promoters in the six random-scanning mutagenesis libraries of P_*ylb*_, flow cytometry was used to quantify EGFP fluorescence. The overnight cultures of all the recombinant *B*. *subtilis* strains were centrifuged at 5000×*g* for 10 min, and the cells were resuspended in ice-cold 1 × phosphate-buffered saline (PBS) buffer (pH 7.4). Flow cytometry analysis was carried out on a FACSCalibur apparatus (Becton Dickinson Biosciences) using excitation at 488 nm. Three independent replicas were analyzed from each promoter library, and each cytometric measurement was performed on 100,000 cells. The data were acquired and analyzed using the CELLQuest software with defined gates that were measured by tight forward scatter/side scatter light to ensure a homogenous population size. The average relative fluorescence intensity (RFI) given represent the mean values of the geometric mean expression values of each replicate; the standard deviation was also calculated from the replicate geometric mean values. The value of coefficient of variation (CV) for each promoter library was obtained by dividing the standard deviation of all replicate values by the mean of all replicate values.

### Screening of the T2 and T3 mutant libraries

The T2 and T3 mutant libraries were screened for P_*ylb*_ mutants with respectively higher and lower activities by using a BD Influx flow cytometer. The separation gates in the dot plots of the two-parameter histogram were reset beyond the negative control (*B*. *subtilis* WB600) values and the positive control (wild type P_*ylb*_) values. P4 gate was used to collect up-regulated P_*ylb*_ mutants, while the P2 gate collected the extremely weak mutants (Supplementary Fig. 1). The fluorescent intensity per OD_600_ (FI/OD_600_) of these selected mutants were measured by a SpectraMax M2 (Molecular Devices, USA) microplate reader. The excitation/emission was read at 484/507 nm. The promoters of these selected mutants were sequenced on an ABI 3730 DNA analyzer (Qingke Biotechnology Co., Ltd.) with the sequencing primers F4-cx-up and F4-cx-down (Supplementary Table 2). All the sequences were analyzed using the WebLogo online software (https://weblogo.berkeley.edu) to explore the relationship between signature sequences and the strength of P_*ylb*_.

### Construction of site-directed mutagenesis promoters

The promoter P_*ylb*_ in the plasmids P_*ylb*_-G-P43-R-pUBC19, P_*ylb*_-R-pUBC19, P_*ylb*_-ophc2-pUBC19 and P_*ylb*_-katA-pUBC19 were mutated into P_*ylb*_-T2G, P_*ylb*_-T2G and P_*ylb*_-T3G, respectively (Supplementary Table 1). To do this, these four plasmids were used as templates to amplify the fragments containing the promoters P_*ylb*_-T2G, P_*ylb*_-T2G and P_*ylb*_-T3G by the primer pairs P16-T2GF/V-R, P16-T2CF/V-R and P16-T3GF/V-R, and the vector fragments by the primer pairs P16-Fr-2 (P16-Fr-3)/V-F (Supplementary Table 2). The corresponding promoter and vector fragments were assembled through POE-PCR, resulting in the 12 plasmids pGT2G, pGT2C, pGT3C, pRT2G, pRT2C, pRT3C, pOT2G, pOT2C, pOT3C, pKT2G, pKT2C and pKT3C (Supplementary Table 1). The sequences of these mutated plasmids were confirmed by DNA sequencing, and then transformed into *B. subtilis* WB600.

### Determination of the transcription levels of *egfp* by quantitative reverse transcription-PCR (qRT-PCR)

To determine the promoter’s activity, total RNA was isolated from 18 h cultures of the transformed *B*. *subtilis* strains EGFP-WT, EGFP-T2G, EGFP-T2C and EGFP-T3C, harboring the recombinant plasmids P_*ylb*_-G-P43-R-pUBC19, pGT2G, pGT2C, and pGT3C, using a RNAprep PureCell/Bacteria Kit (TIANGEN Biotech, Beijing, China). The first strand of cDNA was synthesized using TransScript One-Step gDNA Removal and cDNA Synthesis SuperMix (TransGen Biotech, Beijing, China) with the gene-specific primers *egfp*-rtR and BS16S-rtR (Supplementary Table 2). The qRT-PCR was performed using the SYBR Green Real-time PCR Master Mix Plus (Toyobo, Osaka, Japan) with the primers *egfp*-rtF and *egfp*-rtR (Supplementary Table 2) for the *egfp* gene and primers BS16S-rtF and BS16S-rtR (Supplementary Table 2) for the *16SrDNA* gene.

### Determination of the expression levels of green fluorescent protein EGFP and red fluorescent protein mApple

To determine the yield of fluorescent protein, the overnight cultures of the recombinant *B*. *subtilis* strains were inoculated into a 96-well microtiter plate containing 200 μL LB liquid medium with 10 μg/mL kanamycin per well, and then incubated at 37 °C with shaking at 750 rpm in an incubator 1000 (Heidolph, Germany) for 20 h. Fluorescence intensity and cell density (OD_600_) were measured using a SpectraMax M2 (Molecular Devices, USA) microplate reader. The excitation/emission was read at 484/507 nm for EGFP and 562/598 nm for mApple. Samples were collected from 20 h cultures for SDS-PAGE analysis.

### Heterologous protein expression and enzyme activity analysis

A fresh overnight culture of the recombinant *B*. *subtilis* strains OPHC2-WT, OPHC2-T2G, OPHC2-T2C, and OPHC2-T3C, KatA-WT, KatA-T2G, KatA-T2C, and KatA-T3C were inoculated into 50 mL LB liquid medium containing 10 μg/mL kanamycin and cultivated at 37 °C, at 200 rpm, for 28 h. Organophosphorus hydrolase (OPHC2) activities were determined as described previously (Yu et al. 2015). One unit of OPHC2 activity was defined as the amount of the enzyme required to liberate 1 μmol of *p*-nitrophenol per minute at 37 °C. For catalase activity, the reaction was performed as described previously (Goldblith and Proctor 1950) and one unit of catalase activity was defined as the amount of enzyme required to catalyze the decomposition of 1 μmol H_2_O_2_ per minute at 30 °C.

## Results

### Construction and characterization of the P_*ylb*_ random-scanning mutagenesis libraries

To identify the key elements that influence the promoter strength in the regions flanking the − 35 and − 10 regions of P_*ylb*_, six mutant promoter libraries, T1–T6, were constructed (Fig. 1a) and EGFP fluorescence was quantified using flow cytometry to determine the *egfp* expression levels. The T2 library had the highest RFI (18.7% increase), while the T3 library had the lowest RFI (67.0% decrease), relative to the wild type P_*ylb*_ (Fig. 1b). Meanwhile, the highest CV was observed in the T3 library (Fig. 1b). These data suggested that the four nucleotides flanking the − 35 box of P_*ylb*_ have the most critical influence on promoter strength and randomized mutations in the T3 locus of P_*ylb*_ were likely to cause more obvious changes in the promoter strength.

### Characterization of P_*ylb*_ mutants collected from the T2 and T3 libraries

**Fig. 2:**
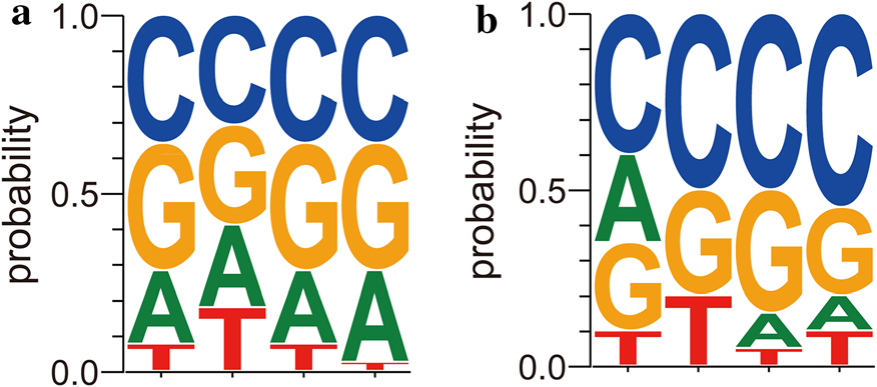
Sequence features of the P_*ylb*_ mutants collected from the **a** T2 and the **b** T3 library; the data was analyzed by the WebLogo online software (https://weblogo.berkeley.edu)

To determine the effect of the four nucleotides flanking the − 35 box of P_*ylb*_ on the promoter activity, the T2 and T3 libraries were selected to sort out the up-regulated and down-regulated variants by flow cytometry and the sequences of these collected promoters were determined by DNA sequencing. 58 clones, of which FI/OD_600_ increased by 30% (Supplementary Fig. 2a), were collected from the T2 library, and these represented 39 different mutated promoters (Supplementary Fig. 3a). 39 clones, with FI/OD_600_ 80% lower than the wild type (Supplementary Fig. 2b), were collected from the T3 library, representing 20 different mutated promoters (Supplementary Fig. 3b). To detect any features of residues at the T2 and T3 locus, all these sequences were subjected to WebLogo analysis. Our findings revealed that the sequences in the T2 and T3 locus of these collected promoter mutants were GC-rich and C-rich, respectively (Fig. 2). These results suggested that the GC bases in the T2 and the C content in the T3 locus were respectively positively and negatively correlated with the transcriptional activity of P_*ylb*_.

### Characterization of the effect of the identified T2 and T3 locus signatures on the promoter activity

**Fig. 3:**
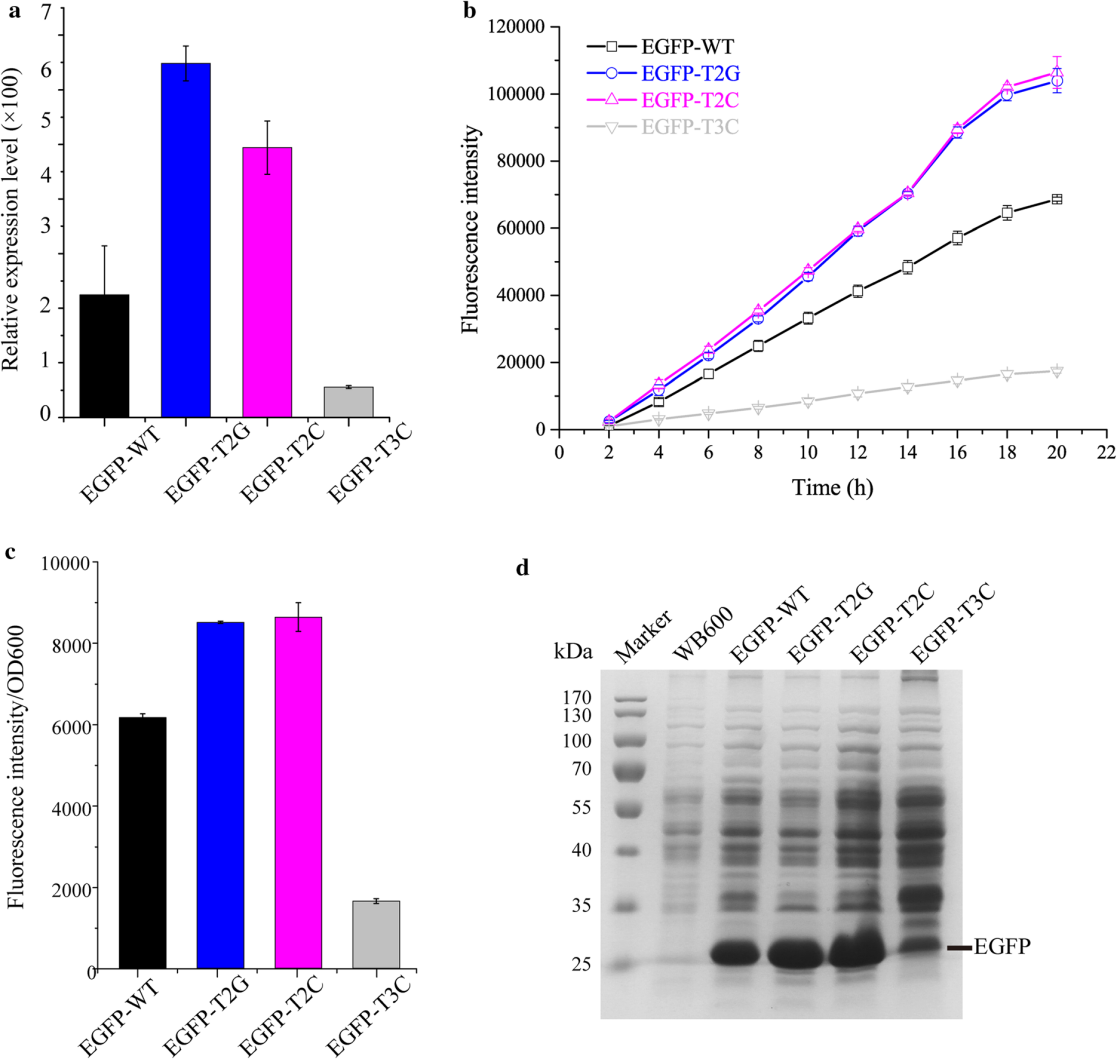
Characterization of P_*ylb*_-T2G, P_*ylb*_-T2C, P_*ylb*_-T3C variants and native P_*ylb*_ using the reporter gene *egfp*. **a** qRT-PCR to analyze the relative expression levels of EGFP under the control of wild type and mutated promoters at 18 h. The *16 S rRNA* gene was used as an internal control for normalization. The data were analyzed by the 2^−ΔΔCt^ method. All the data were independently repeated in triplicates. **b** The yields of total fluorescence intensity (FI) in the *B*. *subtilis* strains harboring P_*ylb*_-G-P43-R-pUBC19 (EGFP-WT), pGT2G (EGFP-T2G), pGT2C (EGFP-T2C) and pGT3C (EGFP-T3C) plasmids during the whole culturing period. **c** The relative fluorescent intensity measured in the EGFP-WT, EGFP-T2G, EGFP-T2C and EGFP-T3C strains, after 20 h of culturing, is represented by the FI per OD_600_. **d** SDS-PAGE analysis of the expression level of EGFP in the EGFP-WT, EGFP-T2G, EGFP-T2C and EGFP-T3C strains after 20 h of culturing

To further test the effect of the GC content in the T2 locus and the C content in T3 locus on the promoter activity, the P_*ylb*_ was mutated into P_*ylb*_-T2G and P_*ylb*_-T2C by replacing “ATAT” in the T2 locus by “GGGG” and “CCCC”, respectively, and into P_*ylb*_-T3C by replacing “TTTT” in the T3 locus by “CCCC”. First, the activities of these three mutant promoters and the native P_*ylb*_ were measured by qRT-PCR. The data showed that the activities of the P_*ylb*_-T2G and P_*ylb*_-T2C were higher than that of P_*ylb*_, while the activities of the P_*ylb*_-T3C were much lower than that of P_*ylb*_ (Fig. 3a). Then, the activities of these promoters were further analyzed by assessing the expression of the reporter gene *egfp* in the plasmids P_*ylb*_-G-P43-R-pUBC19, pGT2G, pGT2C, and pGT3C in the *B*. *subtilis* strain WB600. The expression of EGFP in these strains displayed a continuous increase, and the overall expression levels of EGFP at each time point in the *B*. *subtilis* strains EGFP-T2G and EGFP-T2C were the highest, followed by EGFP-WT, and EGFP-T3C (Fig. 3b). Compared to EGFP-WT, the expression levels of EGFP in the EGFP-T2G and EGFP-T2C mutants after 20 h of culturing increased by 51.5% and 55.1%, respectively, while in the EGFP-T3C it decreased by about 74.6% (Fig. 3b). Meanwhile, the relative fluorescent intensity (FI/OD_600_) was measured and the data showed that the expression levels of EGFP in EGFP-T2G and EGFP-T2C was 1.35-fold and 1.40-fold higher that of EGFP-WT; while the expression levels of EGFP-T3C dropped by 73.2% (Fig. 3c). The final protein expression levels measured by SDS-PAGE were consistent with those measured by fluorescence intensity (Fig. 3d).

**Fig. 4:**
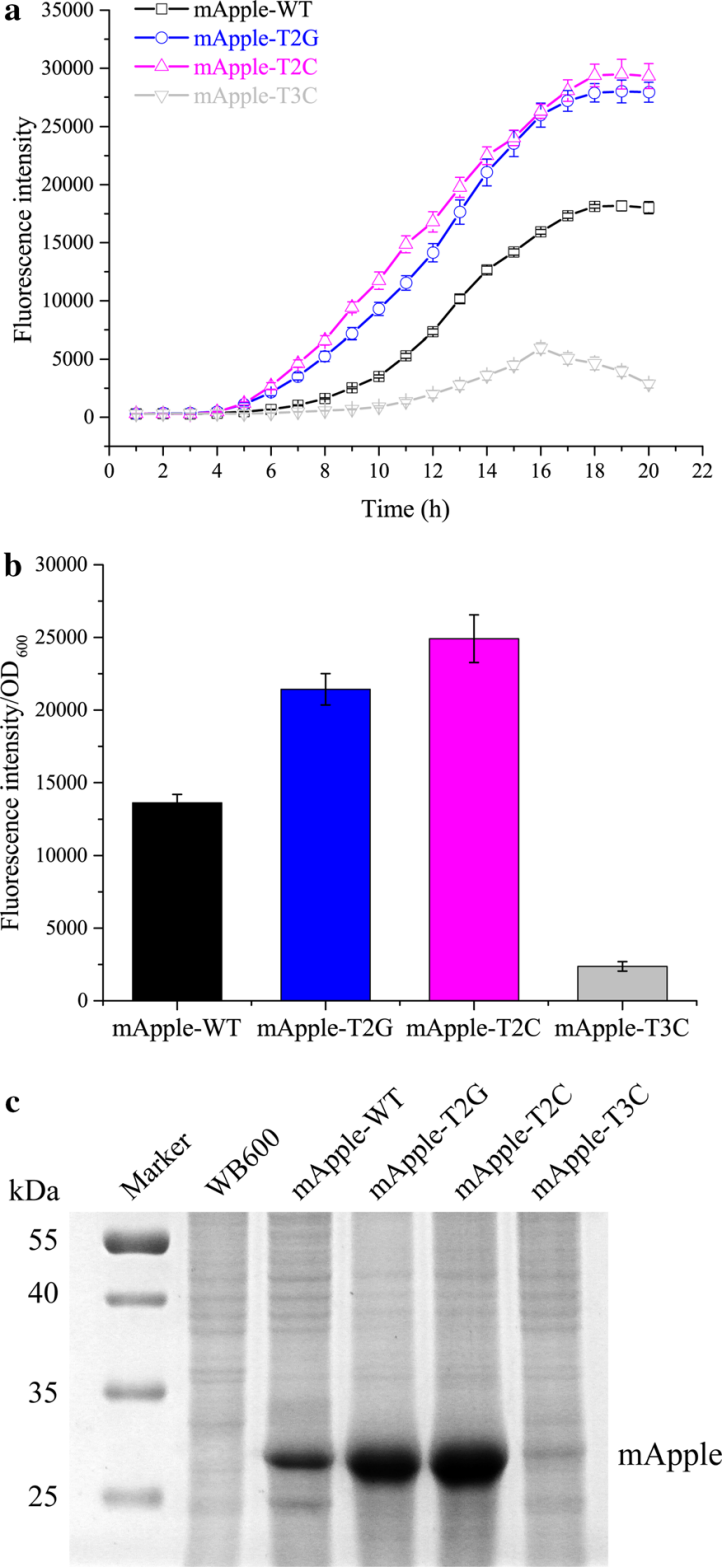
Characterization of the P_*ylb*_-T2G, P_*ylb*_-T2C, P_*ylb*_-T3C variants and native P_*ylb*_ using the reporter gene *mApple*. **a** The total fluorescence intensity yields in the *B*. *subtilis* strains harboring the P_*ylb*_-R-pUBC19 (mApple-WT), pRT2G (mApple-T2G), pRT2C (mApple-T2C) and pRT3C (mApple-T3C) plasmids during the whole culturing period. **b** The relative fluorescent intensity in the mApple-WT, mApple-T2G, mApple-T2C and mApple-T3C strains after 20 h culturing, which is represented by the FI per OD_600_. **c** SDS-PAGE analysis of the expression levels in the mApple in mApple-WT, mApple-T2G, mApple-T2C and mApple-T3C strains after 20 h culturing

To further test the reliability of the signature sequences, the three mutant promoters P_*ylb*_-T2G, P_*ylb*_-T2C, P_*ylb*_-T3C and the native P_*ylb*_ were used to express the reporter gene *mApple* using the plasmids pRT2G, pRT2C, pRT3C and P_*ylb*_-R-pUBC19 in the *B*. *subtilis* strain WB600. These three mutated promoters have similar regulatory trends between the expression of mApple and EGFP (Fig. 4). The expression levels of mApple in the mApple-T2G and mApple-T2C was respectively 1.57-fold and 1.83-fold higher than that in the mApple-WT, while the expression levels of mApple in the mApple-T3C decreased by 82.6% (Fig. 4b).

The above results supported that the extracted signature sequences in the T2 and T3 locus could differentially regulate the activity of the P_*ylb*_ promoter.

### The application prospect of the signature sequences

**Fig. 5:**
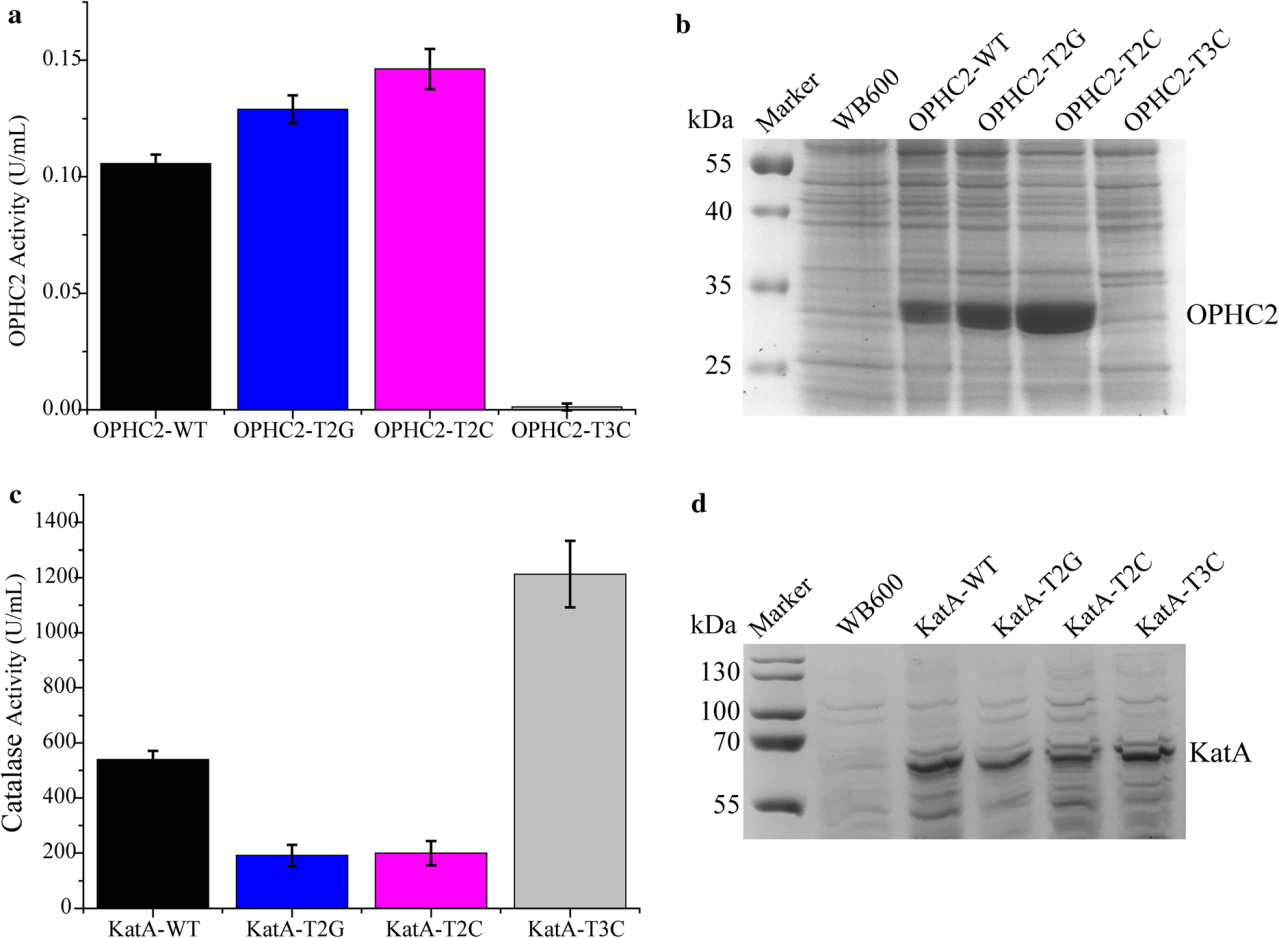
Expression of organophosphorus hydrolase OPHC2 and catalase KatA in the recombinant *B*. *subtilis* strain under the control of the variant promoters P_*ylb*_-T2G, P_*ylb*_-T2C, P_*ylb*_-T3C and the native promoter P_*ylb*_. **a** The enzymatic activity yields of OPHC2 in the *B*. *subtilis* strains harboring the P_*ylb*_-ophc2-pUBC19 (OPHC2-WT), pOT2G (OPHC2-T2G), pOT2C (OPHC2-T2C) and pOT3C (OPHC2-T3C) plasmids. **b** SDS-PAGE analysis of the expression levels of the OPHC2 in the OPHC2-WT, OPHC2-T2G, OPHC2-T2C and OPHC2-T3C strains. **c** The enzymatic activity yields of KatA in the *B*. *subtilis* strains harboring the P_*ylb*_-katA-pUBC19 (KatA-WT), pKT2G (KatA-T2G), pKT2C (KatA-T2C) and pKT3C (KatA-T3C) plasmids. **d** SDS-PAGE analysis of the expression levels of the catalase in the KatA-WT, KatA-T2G, KatA-T2C and KatA-T3C strain

To evaluate the applicability of the signature sequences, the three mutant promoters P_*ylb*_-T2G, P_*ylb*_-T2C, P_*ylb*_-T3C and the native P_*ylb*_ were used to express organophosphorus hydrolase OPHC2 in the *B*. *subtilis* strains OPHC2-T2G, OPHC2-T2C, OPHC2-T3C and OPHC2-WT, and catalase KatA in the *B*. *subtilis* strains KatA-T2G, KatA-T2C, KatA-T3C and KatA-WT. The OPHC2 activities detected in strain OPHC2-T2G and OPHC2-T2C were 22% and 38% higher than that of strain OPHC2-WT, while there was no obvious enzyme activity in the OPHC2-T3C (Fig. 5a). The catalase activities detected in strain KatA-T2G and KatA-T2C were about 63% lower than that of strain KatA-WT, while the catalase activity detected in strain KatA-T3G was 125% higher than that of strain KatA-WT (Fig. 5c). The results of SDS-PAGE analysis of OPHC2 and KatA in the culture supernatant were shown in Fig. 5b and d. These data indicated that the strong promoters P_*ylb*_-T2G, P_*ylb*_-T2C were more suitable for overexpression of OPHC2, whereas, the weak promoter P_*ylb*_-T3C was more suitable for overexpression of KatA. This means that the signature sequences could be used to tune the promoter strength to meet the requirement of expressing different proteins.

## Discussion

Promoters are important regulatory elements for expressing protein. To date, some high-quality native promoters have been widely used in *B*. *subtilis* for gene expression. However, most of the endogenous promoters in *B*. *subtilis* were not well explored and comprehensively analyzed. The promoter P_*ylb*_ was found and proved to be a highly active promoter of *B*. *subtilis* in our lab (Yu et al. 2015). In this study, for better application of the promoter P_*ylb*_, the elements that influence its activity were researched.

One commonly used strategy for exploring promoter element is to randomly mutate within the whole promoter sequence. It is time-consuming and easily missing information. Our precious work shown that the promoter regions I (position − 38 to − 23, relative to TSS) and II (position − 18 to − 3, relative to TSS) that contain the − 35 and the − 10 regions were particularly important for the transcriptional activity of P_*ylb*_ (Yu et al. 2015). Therefore, we focused on the sequence flanking the − 35 and the − 10 regions to identify and characterize elements, apart from the core elements, which influence the promoter strength. In our study, the sequence to be interested in were divided into six groups and each group contained four nucleotides (Fig. 1a), so that each random mutagenesis library potentially contained 256 mutant promoters. Meanwhile, the transformation efficiency of the WB600 strain was 768 cfu/μg. Thus, the transformants were predicted to cover more than 99% of the possible mutants. Together with the high-throughput screening by flow cytometry, our methodological approach could thoroughly explore signature elements with higher efficiency, as compared to the traditional methods of studying promoter activity by random mutagenesis within the whole promoter sequence. Thus it can be used to systematically determine the promoter sequence features which were influenced transcriptional activity in *B. subtilis*.

Previous studies have demonstrated that the spacer region between the − 35 and the − 10 box within the bacterial promoter is critical to regulate the expression levels of heterologous genes (Guiziou et al. 2016; Han et al. 2017; Jensen and Hammer 1998). Our work demonstrated that the GC content in the sequence flanking the − 35 box in the T2 locus enhanced the promoter activity, while the C content in the T3 locus reduced the promoter activity. However, the rationale for this influence in transcriptional activities is obscure. As the -35 element is responsible for recruiting RNA polymerase (Hawley and McClure 1983), the GC content in the four nucleotides flanking the − 35 box may impact RNA polymerase recruiting. This could involve higher-level regulatory effects like DNA looping (Cournac and Plumbridge 2013).

A promoter with a single characteristic is unlikely to satisfy each exogenous gene since, for example, strong overexpression is not always optimal for every given gene. We can see that, the strong promoters P_*ylb*_-T2G and P_*ylb*_-T2C weren’t suitable for the expression of catalase KatA. As the catalase KatA in *B. subtilis* is an extracellular enzyme without known signal peptide (Naclerio et al. 1995), it may be secreted via non-classical secretion pathways. However, the mechanisms of non-classical secretion are unidentified. We speculated that the catalase KatA expressed by the strong promoter could not be folded correctly in time, which affected the secretion, resulting in the decreased extracellular expression.

The T2 and T3 libraries included mutant promoters that induced variable transcriptional levels, ranging from high to low (Supplementary Fig. 4). The randomization of the four nucleotides flanking the − 35 box gave rise to dynamic expression that covered a wide range of levels, so every promoter with differential activity in the T2 and T3 libraries could be potentially used to express genes that require the corresponding transcriptional activity. Thus, the T2 and T3 libraries have great application potentials for enzyme production, metabolic engineering, and synthetic biology.

In conclusion, our thorough characterization of the P_*ylb*_ sequence provides further information for the potential application of this promoter, and contributes to the enrichment of the *B*. *subtilis* promoter library*.* Particularly, the T2 and T3 libraries cover a wide range of promoters with dynamic activities which have great potential to be applied for highly effective tuning of gene expression and regulation of complex metabolic pathways.

## Electronic supplementary material

Below is the link to the electronic supplementary material.

Supplementary file1 (DOCX 806 kb)

## References

[CR1] Bhavsar AP, Zhao X, Brown ED (2001). Development and characterization of a xylose-dependent system for expression of cloned genes in *Bacillus subtilis*: conditional complementation of a teichoic acid mutant. Appl Environ Microbiol.

[CR2] Blazeck J, Alper HS (2013). Promoter engineering: recent advances in controlling transcription at the most fundamental level. Biotechnol J.

[CR3] Cheng J, Guan C, Cui W, Zhou L, Liu Z, Li W, Zhou Z (2016). Enhancement of a high efficient autoinducible expression system in *Bacillus subtilis* by promoter engineering. Protein Expr Purif.

[CR4] Cournac A, Plumbridge J (2013). DNA looping in prokaryotes: experimental and theoretical approaches. J Bacteriol.

[CR5] De Mey M, Maertens J, Lequeux GJ, Soetaert WK, Vandamme EJ (2007). Construction and model-based analysis of a promoter library for *E. coli*: an indispensable tool for metabolic engineering. BMC Biotechnol.

[CR6] Goldblith SA, Proctor BE (1950). Photometric determination of catalase activity. J Biol Chem.

[CR7] Guan C, Cui W, Cheng J, Zhou L, Liu Z, Zhou Z (2016). Development of an efficient autoinducible expression system by promoter engineering in *Bacillus subtilis*. Microb Cell Fact.

[CR8] Guiziou S, Sauveplane V, Chang HJ, Clerte C, Declerck N, Jules M, Bonnet J (2016). A part toolbox to tune genetic expression in *Bacillus subtilis*. Nucleic Acids Res.

[CR9] Han LC (2017). Fabrication and characterization of a robust and strong bacterial promoter from a semi-rationally engineered promoter library in *Bacillus subtilis*. Process Biochem.

[CR10] Hawley DK, McClure WR (1983). Compilation and analysis of *Escherichia coli* promoter DNA sequences. Nucleic Acids Res.

[CR11] Jensen PR, Hammer K (1998). The sequence of spacers between the consensus sequences modulates the strength of prokaryotic promoters. Appl Environ Microbiol.

[CR12] Lee SJ, Pan JG, Park SH, Choi SK (2010). Development of a stationary phase-specific autoinducible expression system in *Bacillus subtilis*. J Biotechnol.

[CR13] Ming YM, Wei ZW, Lin CY, Sheng GY (2010). Development of a *Bacillus subtilis* expression system using the improved P_*glv*_ promoter. Microb Cell Fact.

[CR14] Naclerio G, Baccigalupi L, Caruso C, De Felice M, Ricca E (1995). *Bacillus subtilis* vegetative catalase is an extracellular enzyme. Appl Environ Microbiol.

[CR15] Phan TT, Nguyen HD, Schumann W (2012). Development of a strong intracellular expression system for *Bacillus subtilis* by optimizing promoter elements. J Biotechnol.

[CR16] Phan TT, Tran LT, Schumann W, Nguyen HD (2015). Development of Pgrac100-based expression vectors allowing high protein production levels in *Bacillus subtilis* and relatively low basal expression in *Escherichia coli*. Microb Cell Fact.

[CR17] Spizizen J (1958). Transformation of biochemically deficient strains of *Bacillus subtilis* by deoxyribonucleate. Proc Natl Acad Sc USA.

[CR18] Steinmetz M, Le Coq D, Aymerich S, Gonzy-Treboul G, Gay P (1985). The DNA sequence of the gene for the secreted *Bacillus subtilis* enzyme levansucrase and its genetic control sites. Mol Gen Genet.

[CR19] van Dijl JM, Hecker M (2013). *Bacillus subtilis*: from soil bacterium to super-secreting cell factory. Microb Cell Fact.

[CR20] You C, Percival Zhang YH (2012). Easy preparation of a large-size random gene mutagenesis library in *Escherichia coli*. Anal Biochem.

[CR21] Yu X (2015). Identification of a highly efficient stationary phase promoter in *Bacillus subtilis*. Sci Rep.

[CR22] Zhang XZ, Cui ZL, Hong Q, Li SP (2005). High-level expression and secretion of methyl parathion hydrolase in *Bacillus subtilis* WB800. Appl Environ Microbiol.

[CR23] Zhou C, Ye B, Cheng S, Zhao L, Liu Y, Jiang J, Yan X (2019). Promoter engineering enables overproduction of foreign proteins from a single copy expression cassette in *Bacillus subtilis*. Microb Cell Fact.

