# Supplementary material for: Identification and characterization of sequence signatures in the Bacillus subtilis promoter Pylb for tuning promoter strength
Source: Biotechnol Lett. 2019 Nov 5;42(1):115–24. doi: 10.1007/s10529-019-02749-4 (PMC6940355; doi:10.1007/s10529-019-02749-4)
Supplement: Supplementary file 1 — Supplementary file1 (DOCX 806 kb) [file 10529_2019_2749_MOESM1_ESM.docx]

**Supplementary information**

**Identification and characterization of sequence signatures** **in the *Bacillus subtilis* promoter P*_ylb_* for tuning promoter strength**

**Jiangtao Xu****·Xiaoqing Liu·Xiaoxia Yu·Xiaoyu Chu·Jian Tian·Ningfeng Wu**

Jiangtao Xu and Xiaoqing Liu have contributed equally to this work

J. Xu·X. Liu·X. Yu·X. Chu·J. Tian (
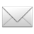
)·Ningfeng Wu (
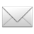
)

Biotechnology Research Institute, Chinese Academy of Agricultural Sciences, Beijing 100081, China

e-mail: tianjian@caas.cn

N. Wu

e-mail: wuningfeng@caas.cn

**Table 1** **Plasmids and strains used in this study**

| **Plasmids and strains** | **Description** | **Source** |
| --- | --- | --- |
| **Plasmids** |  |  |
| pUBC19 | *E*. *coli*-*B*. *subtilis* shuttle plasmid, *Amp^r^*, *Kana^r^* | Lab stock |
| R-G-pUBC19 | derived from pUBC19, integration within reports, *mApple* (R) and *egfp* | (Yu et al. 2015) |
| P*_ylb_*-G-P43-R-pUBC19 | derived from R-G-pUBC19, binary vector in which *egfp* was trigged by P*_ylb_* while *mApple* was controlled by P43 | (Yu et al. 2015) |
| pGT2G | T2 position ATAT of P*_ylb_* was replaced by GGGG in P*_ylb_*-G-P43-R-pUBC19 | This study |
| pGT2C | T2 position ATAT of P*_ylb_* was replaced by CCCC in P*_ylb_*-G-P43-R-pUBC19 | This study |
| pGT3C | T3 position ATAT of P*_ylb_* was replaced by CCCC in P*_ylb_*-G-P43-R-pUBC19 | This study |
| P*_ylb_*-R-pUBC19 | derived from pUBC19, *mApple* was controlled by P*_ylb_* | Lab stock |
| pRT2G | T2 position ATAT of P*_ylb_* was replaced by GGGG in P*_ylb_*-R-pUBC19 | This study |
| pRT2C | T2 position ATAT of P*_ylb_* was replaced by CCCC in P*_ylb_*-R-pUBC19 | This study |
| pRT3C | T3 position ATAT of P*_ylb_* was replaced by CCCC in P*_ylb_*-R-pUBC19 | This study |
| P*_ylb_*-ophc2-pUBC19 | derived from pUBC19, *ophc2* was controlled by P*_ylb_* | Lab stock |
| pOT2G | T2 position ATAT of P*_ylb_* was replaced by GGGG in P*_ylb_*-ophc2-pUBC19 | This study |
| pOT2C | T2 position ATAT of P*_ylb_* was replaced by CCCC in P*_ylb_*-ophc2-pUBC19 | This study |
| pOT3C | T3 position ATAT of P*_ylb_* was replaced by CCCC in P*_ylb_*-ophc2-pUBC19 | This study |
| P*_ylb_*-katA-pUBC19 | derived from pUBC19, *katA* was controlled by P*_ylb_* | Lab stock |
| pKT2G | T2 position ATAT of P*_ylb_* was replaced by GGGG in P*_ylb_*-katA-pUBC19 | This study |
| pKT2C | T2 position ATAT of P*_ylb_* was replaced by CCCC in P*_ylb_*-katA-pUBC19 | This study |
| pKT3C | T3 position ATAT of P*_ylb_* was replaced by CCCC in P*_ylb_*-katA-pUBC19 | This study |
| **Strains** |  |  |
| *E*. *coli* DH5α | F^-^φ80 *lac*ZΔM15 Δ(lacZYA-arg F) U169, *end*A1, *rec*A1, *hsd*R17(rk-mk+), *sup*E44λ^-^, *thi*-1, *gyr*A96, r*el*A1, *pho*A | TransGene |
| *B*. *subtilis* WB600 | \| *apr, nprA, epr, bpf, mpr, nprB, trpC2* \| \| --- \| | Lab stock |
| EGFP-WT | *B*. *subtilis* WB600 harboring plasmid P*_ylb_*-G-P43-R-pUBC19 | This study |
| EGFP-T2G | *B*. *subtilis* WB600 harboring plasmid pGT2G | This study |
| EGFP-T2C | *B*. *subtilis* WB600 harboring plasmid pGT2C | This study |
| EGFP-T3C | *B*. *subtilis* WB600 harboring plasmid pGT3C | This study |
| mApple-WT | *B*. *subtilis* WB600 harboring plasmid P*_ylb_*-R-pUBC19 | This study |
| mApple-T2G | *B*. *subtilis* WB600 harboring plasmid pRT2G | This study |
| mApple-T2C | *B*. *subtilis* WB600 harboring plasmid pRT2C | This study |
| mApple-T3C | *B*. *subtilis* WB600 harboring plasmid pRT3C | This study |
| OPHC2-WT | *B*. *subtilis* WB600 harboring plasmid P*_ylb_*-ophc2-pUBC19 | This study |
| OPHC2-T2G | *B*. *subtilis* WB600 harboring plasmid pOT2G | This study |
| OPHC2-T2C | *B*. *subtilis* WB600 harboring plasmid pOT2C | This study |
| OPHC2-T3C | *B*. *subtilis* WB600 harboring plasmid pOT3C | This study |
| KatA-WT | *B*. *subtilis* WB600 harboring plasmid P*_ylb_*-katA-pUBC19 | This study |
| KatA-T2G | *B*. *subtilis* WB600 harboring plasmid pCT2G | This study |
| KatA-T2C | *B*. *subtilis* WB600 harboring plasmid pCT2C | This study |
| KatA-T3C | *B*. *subtilis* WB600 harboring plasmid pCT3C | This study |

**Table S2** **Primers used in this study**

| **Primer** | **Sequence (5’-3’)** |
| --- | --- |
| P16-F-1 | GATCCCATGTGCTTAAAATTAAAGT**NNNN**ATATTTGGATTTTTTAAATAAAGCG |
| P16-F-2 | CATGTGCTTAAAATTAAAGTTTAA**NNNN**TTGGATTTTTTAAATAAAGCGTTTA |
| P16-F-3 | AAATTAAAGTTTAAATATTTGGATT**NNNN**AAATAAAGCGTTTACAATATATGTA |
| P16-F-4 | TAAAGTTTAAATATTTGGATTTTTT**NNNN**AAAGCGTTTACAATATATGTAGAAA |
| P16-F-5 | GTTTAAATATTTGGATTTTTTAAAT**NNNN**CGTTTACAATATATGTAGAAACAAC |
| P16-F-6 | AAATATTTGGATTTTTTAAATAAAG**NNNN**TACAATATATGTAGAAACAACAAAG |
| V-R | CTCTCGGTTATGAGTTAGTTCAAATTCGTTCTT |
| P16-Fr-1 | ACTTTAATTTTAAGCACATGGGATC |
| P16-Fr-2 | TTAAACTTTAATTTTAAGCACATG |
| P16-Fr-3 | AATCCAAATATTTAAACTTTAATTTTAAGC |
| P16-Fr-4 | AAAAAATCCAAATATTTAAACTTTAATTTTAAG |
| P16-Fr-5 | ATTTAAAAAATCCAAATATTTAAACTTTAAT |
| P16-Fr-6 | CTTTATTTAAAAAATCCAAATATTTAAACT |
| V-F | TGAACTAACTCATAACCGAGAG |
| F4-cx-up | GATACACGAAATCACGGCAAAAACGCAAAC |
| F4-cx-down | CGCCAGCTGAACAGAGCCATCTTCGATGT |
| P16-T2GF | CATGTGCTTAAAATTAAAGTTTAA**GGGG**TTGGATTTTTTAAATAAAGCGTTTA |
| P16-T2CF | CATGTGCTTAAAATTAAAGTTTAA**CCCC**TTGGATTTTTTAAATAAAGCGTTTA |
| P16-T3CF | AAATTAAAGTTTAAATATTTGGATT**CCCC**AAATAAAGCGTTTACAATATATGTA |
| *egfp*-rtF | GAACTTGATGGTGATGTG |
| *egfp*-rtR | CGTTGTTACTAATGTCGG |
| BS16S-rtF | GTAACCTGCCTGTAAGAC |
| BS16S-rtR | CATCTGTAAGTGGTAGCC |

**
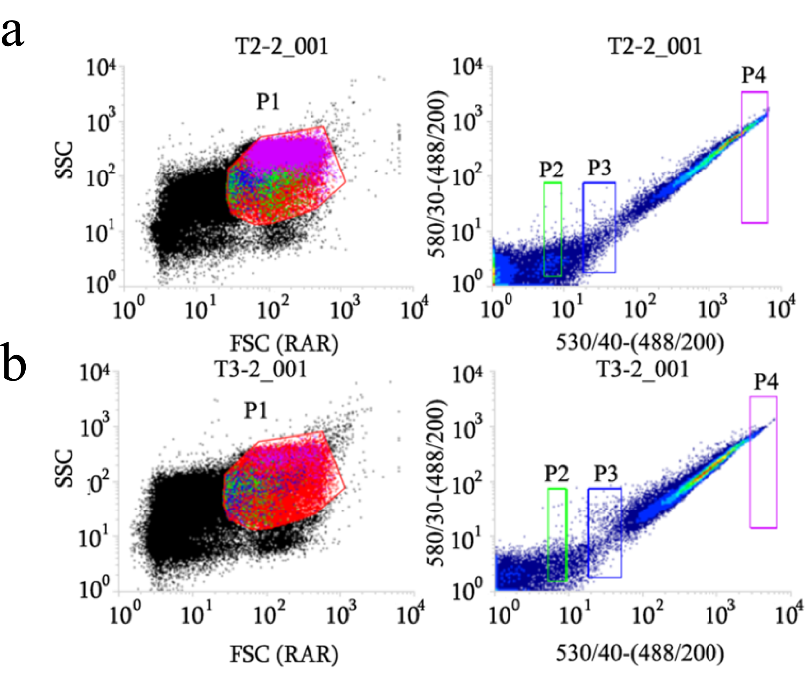
**

**Fig. 1** Cell sorting of *B*. *subtilis* mutant strains from the (**a**) T2 and (**b**) T3 randomly mutated promoter libraries. P4 gate was used to collect up-regulated P*_ylb_* mutants, while the P2 gate collected the extremely weak mutants


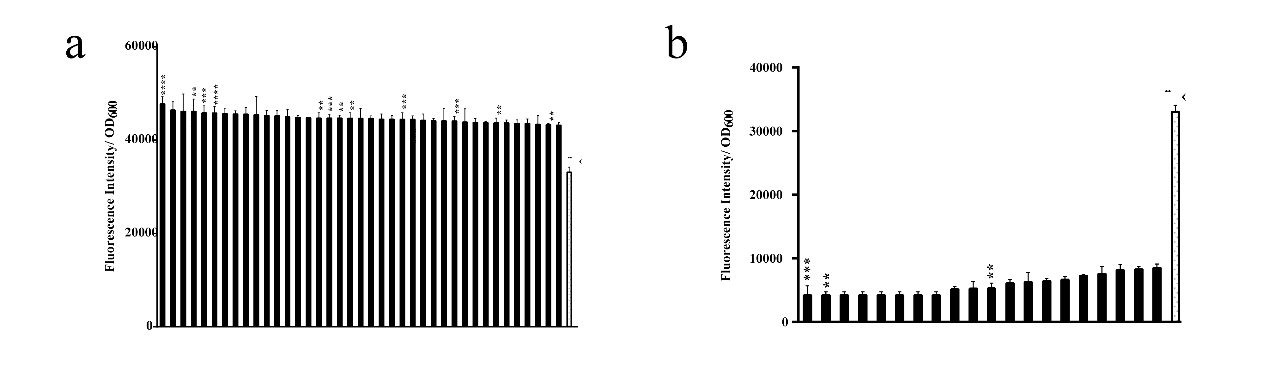


**Fig. 2** The relative fluorescent intensity of EGFP driven by selected promoter variants of P*_ylb_* from the (**a**) T2 and (**b**) T3 libraries. The column shown in gray is the relative FI of the native P*_ylb_*, and the columns shown in black are the relative FI of the mutated variants of P*_ylb_*. The number of the asterisk (*) represents the promoter sequence number

**
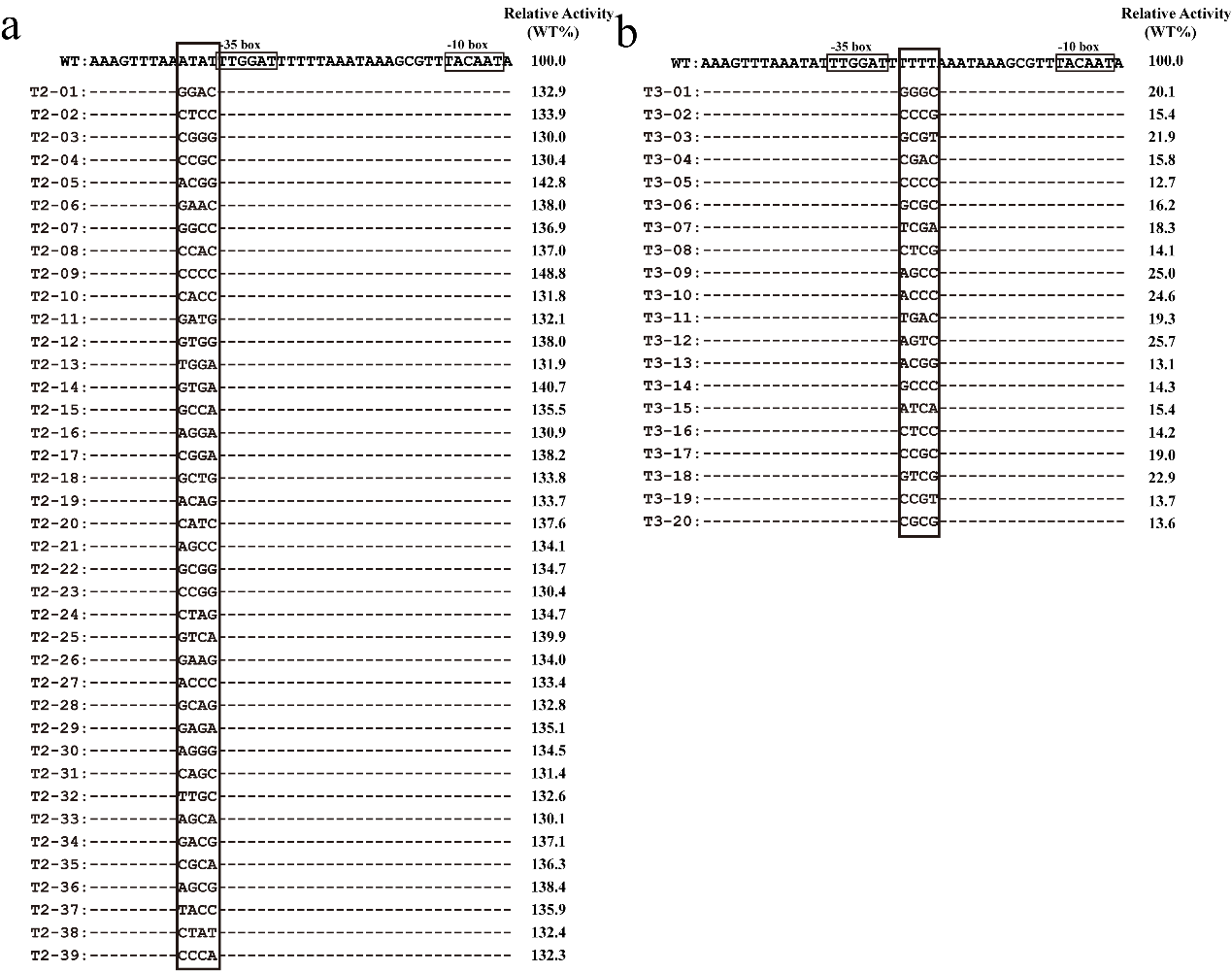
**

**Fig. 3** Sequences of the P*_ylb_* mutants collected from the (**a**) T2 and the (**b**) T3 library

**
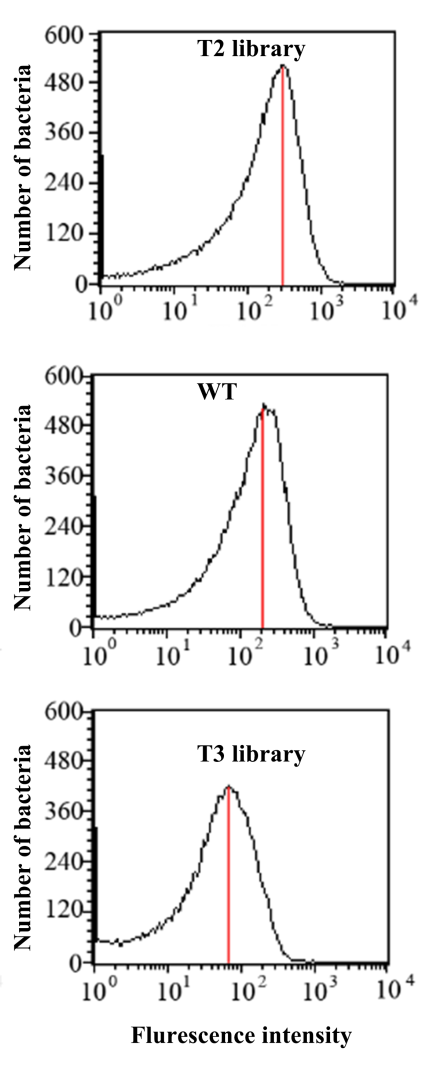
**

**Fig. 4** Distribution of the expression levels of EGFP driven by promoter variants from the T2 and T3 libraries. Histograms were plotted with the values obtained by measurements of fluorescence intensity in the pool of variants by flow cytometry

**The DNA sequence of the *egfp* gene**

atggtgtccaaaggtgaagaactgtttacgggtgtagtgcctatcctggttgaacttgatggtgatgtgaacggtcataaattttccgtatccggcgaaggagaaggtgatgctacatatggcaaactgacgcttaaatttatttgcacaacgggaaaacttccggtcccttggccgacattagtaacaacgttgacgtatggagtgcaatgtttttcccgttaccctgatcatatgaaacagcatgatttctttaaatcagctatgccggaaggatacgtccaagaaagaacaattttctttaaagatgacggtaactacaaaacacgcgccgaagttaaatttgaaggtgatacgctggtgaatcgtatcgaacttaaaggcatcgattttaaagaagacggtaacatcctgggccataaacttgaatacaactacaacagccataacgtctacatcatggcagataaacagaaaaatggaatcaaagtaaactttaaaatccggcataacatcgaagatggctctgttcagctggcggaccattatcaacagaatacacctattggcgatggacctgttctgcttccggacaaccattacttatcaacgcagagcgcgttgtctaaagatccgaacgaaaaaagagaccatatggttttattggaatttgtgacagcagcgggaatcacgttaggtatggatgaattgtataaataa

**References**

Yu X et al. (2015) Identification of a highly efficient stationary phase promoter in *Bacillus subtilis*. Sci Rep 5:18405. <https://doi:10.1038/srep18405>
